# TNF-alpha neutralizing antibody blocks thermal sensitivity induced by compound 48/80-provoked mast cell degranulation

**DOI:** 10.12688/f1000research.2-178.v2

**Published:** 2013-09-27

**Authors:** Devavani Chatterjea, Luisa Paredes, Tijana Martinov, Evelyn Balsells, Juliann Allen, Akilah Sykes, Alyssa Ashbaugh

**Affiliations:** 1 Biology Department, Macalester College, St. Paul, MN 55015, USA

## Abstract

**Background:** Neuro-inflammatory circuits in the tissue regulate the complex pathophysiology of pain. Protective nociceptive pain serves as an early warning system against noxious environmental stimuli. Tissue-resident mast cells orchestrate the increased thermal sensitivity following injection of basic secretagogue compound 48/80 in the hind paw tissues of ND4 mice. Here we investigated the effects of pre-treatment with TNF-α neutralizing antibody on compound 48/80-provoked thermal hyperalgesia.

**Methods:** We treated ND4 Swiss male mice with intravenous anti-TNF-α antibody or vehicle 30 minutes prior to bilateral, intra-plantar compound 48/80 administration and measured changes in the timing of hind paw withdrawal observed subsequent to mice being placed on a 51oC hotplate. We also assessed changes in tissue swelling, TNF-α gene expression and protein abundance, mast cell degranulation, and neutrophil influx in the hind paw tissue.

**Findings:** We found that TNF-α neutralization significantly blocked thermal hyperalgesia, and reduced early tissue swelling. TNF-α neutralization had no significant effect on mast cell degranulation or neutrophil influx into the tissue, however. Moreover, no changes in TNF-α protein or mRNA levels were detected within 3 hours of administration of compound 48/80.

**Interpretation:**  The neutralizing antibodies likely target pre-formed TNF-α including that stored in the granules of tissue-resident mast cells. Pre-formed TNF-α, released upon degranulation, has immediate effects on nociceptive signaling prior to the induction of neutrophil influx. These early effects on nociceptors are abrogated by TNF-α blockade, resulting in compromised nociceptive withdrawal responses to acute, harmful environmental stimuli.

## Introduction

Pain is one of the cardinal components of inflammation and tissue-resident immune cells are important players in the regulation of protective nociceptive responses ^1^. Compound 48/80 (c48/80)-provoked thermal hyperalgesic responses in the hind paw tissue of male ND4 Swiss mice are mediated by the degranulation of tissue-resident mast cells ^2^. The increased sensitivity to thermal stimulus is dependent, in part, on neutrophil influx into the affected tissue, and on histamine signaling; the response is completely abrogated in mast cell-deficient mice, and is substantially reduced by blocking neutrophil influx or treatment with histamine receptor antagonists ^2^. The importance of neutrophil influx in nociceptive cascades has been shown in different rodent models ^3– 5^ and the important regulatory roles of inflammatory cytokines including IL-1β, TNF-α, and IL-6 in pain signaling have also been demonstrated ^6, 7^. Here we show that c48/80-induced early thermal sensitivity in ND4 Swiss mice is markedly reduced by pre-treatment with a neutralizing antibody against TNF-α. Anti-TNF-α antibody administration does not significantly affect levels of mast cell degranulation or neutrophil influx into the affected tissue. We also found no increase in protein and mRNA levels of TNF-α in the tissue within the first 3 hours following c48/80 administration suggesting that this blockade targets pre-formed TNF-α including that stored in the granules of tissue-resident mast cells. Our data suggest that pre-formed TNF-α may act rapidly on nociceptors that are known to reside in close proximity to mast cells ^8^ and modulate nociceptor sensitization thresholds ^9^ and function ^10^. TNF-α blockade therefore compromises the early, protective nociceptive withdrawal responses that normally act to protect tissues from further exposure to acute, injurious environmental stimuli ^11^.

## Methods

### Animals

Three-six month old male ND4 Swiss mice (Harlan Laboratories, Indianapolis, IN) were housed in Macalester College’s research animal facility with a 12-hour light/dark cycle and free access to food and water. A total of 73 mice were used for the experiments shown here (21 controls; 52 experimental) to ensure that appropriate statistical analysis could be performed on data acquired from these experiments. Macalester College’s Institutional Animal Care and Use Committee approved all experimental procedures (Protocol B08Su1).

### Drug administration

All drugs were administered using 0.9% saline (VWR, Radnor, PA) vehicle or phosphate buffered saline (EMD Millipore, Billerica, MA). Mice received bilateral intra-plantar (i.pl.) treatments with c48/80 (1.5μg/paw; 10μl; (Sigma-Aldrich, St. Louis, MO)) or saline alone as previously described ^2^. Either 200μg/kg of anti-TNF-α neutralizing antibody (R&D Systems, Minneapolis, MN, Polyclonal Goat IgG, Catalog #: AB-410-NA) or 200μl vehicle was injected intravenously 30 minutes prior to c48/80 injection in a protocol adapted from Rocha *et al.* ^12^. Rocha *et al.* administered anti-TNF-α antibodies i.v. 5 mins prior to carrageenan treatment in a mouse model of mechanical hyperalgesia ^12^.

### Thermal sensitivity assessment

To assess thermal sensitivity, single mice treated with i.pl. c48/80 or vehicle were placed in a Plexiglas cylinder on a hotplate analgesia meter (Harvard Laboratories, Edenbridge, KY) maintained at 51.0 ± 0.5°C and removed when prolonged retraction, flipping/licking of the hind paw, or jumping with both hind paws off the hotplate were observed, but no later than 40 seconds, as previously described ^2^. Two baseline hotplate latencies were taken 24 and 48 hours before the experiment. Mice with >10 second differences between baselines or <15 second averages were excluded from the experiment. Nociceptive thermal sensitivity was quantified by subtracting the mean baseline thermal latency from the experimental thermal latency at each time point for each mouse.

### Paw edema measurements

Change in hind paw width measured using digital calipers (±0.1mm; VWR, Radnor, PA) was calculated as an average of the left and right paw widths. Baseline paw widths for each mouse were taken pre-treatment and subtracted from post-treatment paw widths to calculate tissue edema as previously described ^2^.

### Myeloperoxidase measurements

Footpads were extracted from hind paws of mice euthanized by CO _2_ inhalation, weighed, frozen at -80°C in 5.6μl/mg tissue weight of 50mM K _2_HPO _4_ buffer (pH 6.0) (Sigma-Aldrich, St. Louis, MO) containing 0.05% hexadecyltrimethylammonium bromide (HTAB) (Sigma-Aldrich, St. Louis, MO), thawed, homogenized in 5x the storage volume of HTAB buffer, sonicated with a 550 Sonic Dismembrator (Fisher Scientific, Waltham, MA), freeze-thawed, and centrifuged (AllegraX-15R; Beckman Coulter, Inc., Pasadena, CA) as previously described ^2, 13^. Absorbance was recorded using a BioTek PowerWave XS plate reader (BioTek, Winooski, VT) at 450nm after a 20-minute incubation in 50mM phosphate buffer (pH 6.0) with 0.025% hydrogen peroxide (Sigma Aldrich, St. Louis, MO) and 0.167mg/ml *o*-dianisidine-dihydrochloride (Sigma Aldrich, St. Louis, MO) at room temperature in the dark. Myeloperoxidase (MPO) levels were normalized to tissue weight and presented as OD/g of wet tissue.

### TNF-α measurements

For protein and gene expression studies, hind paws were excised from mice euthanized by CO _2_ inhalation, flash-frozen in liquid nitrogen and stored at -80°C.

For protein studies, flash-frozen paws were homogenized in Cell Lysis Buffer (Cell Signaling Technology, Beverly, MA) supplemented with protease inhibitor (Cocktail Set IV; EMD Biosciences, Billerica, MA) using a Tissue-Tearor (BioSpec; Model 985370). Homogenates were incubated on ice for 20 minutes, centrifuged at 2000 rpm for 10 minutes at 4°C, and lysate supernatants stored at -80°C. We quantified TNF-α cytokine levels by ELISA according to the manufacturer’s instructions (eBioscience, San Diego, CA).

To measure cytokine gene expression, total RNA was extracted from flash-frozen plantar tissue (Total RNA Mini Kit, Midwest Scientific, St. Louis, MO), quantified with Nanodrop ND-1000 Spectrophotometer (Thermo Scientific, Wilmington, DE), and reverse-transcribed in a 2720 Thermal Cycler (Life Technologies, Grand Island, NY) using the Superscript III First-Strand Synthesis System (Life Technologies, Grand Island, NY) with 100ng of RNA per reaction. Relative transcript abundance was determined by quantitative comparative reverse transcriptase PCR (qRT-PCR) ^14^ using TaqMan Gene Expression Assay Primer/Probe Sets and TaqMan MasterMix (Life Technologies, Grand Island, NY) in a Bio-Rad iCycler (Bio-Rad, Hercules, CA) using β-2-microglobulin (β2m; Mm00437762_m1, Life Technologies, Grand Island, NY) and TNF-α (Mm00443260_g1, Life Technologies, Grand Island, NY) primer sets. Fold expression was normalized to β-2-microglobulin levels and calculated as previously described ^14, 15^.

### Histology

Excised hind paws (from mice euthanized by CO _2_ inhalation) were fixed in 10% buffered formalin (VWR, Radnor, PA) for 24 hours, transferred to 70% ethanol (Sigma-Aldrich, St. Louis, MO), decalcified for 1–2 weeks in 15% EDTA, hydrated, and embedded in paraffin. 4μm sagittal sections were stained with toluidine blue (Tb) at pH 2.3 for 3 minutes (Sigma-Aldrich, St. Louis, MO) or hematoxylin and eosin (H&E) (Sigma-Aldrich, St. Louis, MO) for the detection of mast cells or neutrophils, respectively, at 400x magnification with ≥3 biological replicates per treatment. Tb-stained mast cells were counted in 10 fields/section and degranulation was scored based on the number of granules observed outside the boundary of the cell: intact (0), mild (1–10), moderate (10–20), and extensive (20+) as previously described ^2^. The same investigator performed all counts and was blinded to treatment groups. Neutrophils were imaged between the heel and the toes on the plantar side of the hind paw section using an Olympus CX21LED light microscope and camera (Olympus Corporation of the Americas, Center Valley, PA).

### Statistical analysis

Data were processed using Microsoft Excel (Redmond, WA) and graphed with PRISM 5.0 (GraphPad, San Diego, CA). All statistical analyses were performed using JMP 9.0 (SAS, Cary, NC). All data are presented as the mean ±SEM. Data were analyzed using one-way ANOVA, and the Tukey-Kramer HSD *post hoc* test, at each time point. Statistical significance was defined as *p*<0.05. Thermal sensitivity and hind paw edema measurements included 15–18 mice per treatment group. Other analyses used 3–9 mice per treatment group. All data represent 2–3 independent experiments.

## Results

### TNF-α neutralizing antibody blocks thermal hyperalgesia and early tissue swelling induced by compound 48/80-provoked mast cell degranulation in the hind paw tissue of ND4 mice

**Figure 1.**
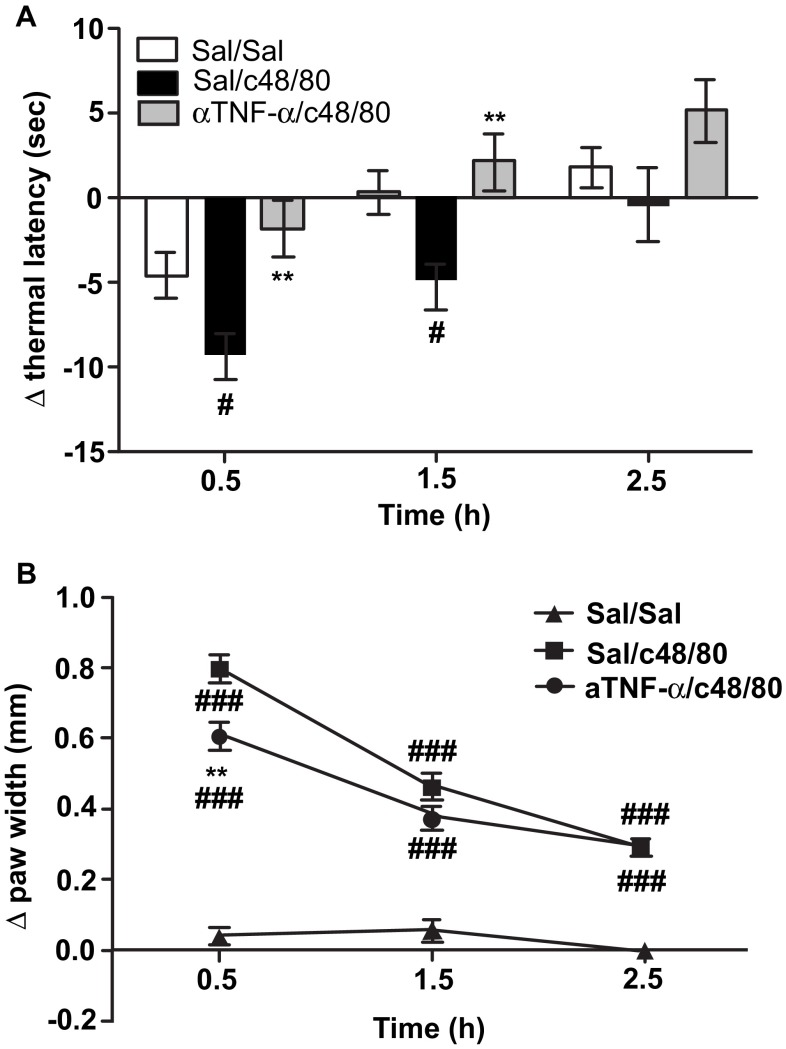
Anti-TNF-α neutralizing antibody abrogates c48/80-induced thermal hyperalgesia. Mice were pre-treated with 200μg/kg anti-TNF-α neutralizing antibody or vehicle (200μl, *i.v.*) 30 minutes before bilateral intra-plantar c48/80 or 0.9% saline injections (1.5μg/paw; 10μl). The bars represent the mean change in thermal paw withdrawal latency ( **A**) and the change in paw width ( **B**) from baseline and error bars represent ± SEM. Anti-TNF-α administration abrogated thermal hyperalgesia at 0.5 and 1.5h after c48/80 injection ( **A**) and reduced paw edema significantly at 0.5h ( **B**). Significances are compared to Sal/Sal (# = *p*<0.05; ## = *p*<0.001) and Sal/c48/80 (* = *p*<0.05; *** = *p*<0.001). n = 12 in Sal/Sal; n = 18 for Sal/c48/80; n = 19 for anti-TNF/c48/80 treatment groups; data are pooled from 2 independent experiments.

Male ND4 Swiss mice bilaterally injected with 1500ng of c48/80 in the hind paws showed hyperalgesic withdrawal responses ~10 seconds sooner than their baseline withdrawal 30 minutes after treatment; these responses were resolved by 2.5 hours ( Figure 1A). Pre-treatment with an intravenous injection of 200μg/kg TNF-α neutralizing antibody 30 minutes prior to c48/80 injection significantly abrogated these responses at 30 minutes and 1.5 hours after intra-plantar treatment with c48/80; pre-treated mice showed hyperalgesic withdrawal responses <5 seconds sooner than their baseline responses 30 minutes after treatment ( Figure 1A). Withdrawal responses of control and anti-TNF-α treated groups were significantly different at 1.5 hours after treatment and indistinguishable at 2.5 hours when the c48/80-provoked hyperalgesic responses were resolved. Anti-TNF-α pretreatment also significantly reduced hind paw edema compared to mice without pre-treatment at 30 minutes but not at 1.5 and 2.5 hours ( Figure 1B). Saline-treated control mice had no increase in thermal sensitivity and showed no tissue swelling ( Figure 1A–B). Overall, thermal sensitivity was reduced in a more sustained manner compared to tissue edema.

**Figure 2.**
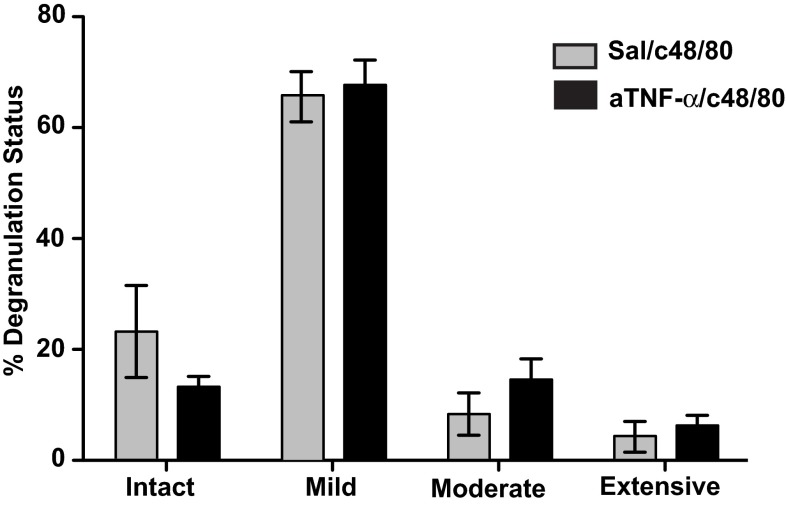
Pre-treatment with anti-TNF-α does not affect c48/80-provoked mast cell degranulation. Mice were pre-treated with 200μg/kg anti-TNF-α neutralizing antibody or vehicle (200μl, *i.v.*) 30 minutes before bilateral intra-plantar c48/80 injection (1.5μg/paw; 10μl). Mice were euthanized 2.5h after c48/80 administration; their hind paws were harvested, preserved in 10% buffered formalin and ethanol, and 4μm paraffin sections stained with toluidine blue for mast cell visualization. Mast cells were counted at 400x and their degranulation status was assessed as described previously ^2^. Bars represent mean percentages of mast cells examined that are assigned to intact or mild, moderate and extensive degranulation status respectively and the error bars represent ±SEM. n = 3 mice per treatment group; data are representative of 2 experiments.

To confirm that the antibody pre-treatment did not have an effect on the extent of mast cell degranulation caused by c48/80 that would have consequently affected mast cell-mediated hyperalgesia, we evaluated the levels of degranulation in toluidine blue-stained 4μm paraffin sections as previously described ^2^ and confirmed that the levels of mild, moderate and extensive degranulation were not altered in anti-TNF-α treated mice ( Figure 2).

Taken together, pre-treatment with anti-TNF-α neutralizing antibody did not change the extent of local mast cell degranulation in the mouse hind paw tissue but markedly reduced resulting thermal sensitivity and tissue swelling provoked by injection of the basic mast cell secretagogue c48/80.

### Intra-plantar c48/80 administration does not induce changes in TNF-α protein or mRNA levels in the hind paw tissue of ND4 male mice

**Figure 3.**
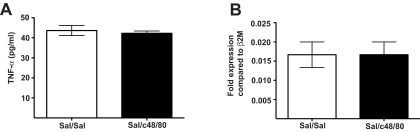
Intra-plantar c48/80 administration does not induce changes in TNF-α protein or mRNA levels in the hind paw tissue of ND4 male mice. Mice were pretreated with 200μg/kg anti-TNF-α neutralizing antibody or vehicle (200μg, *i.v.*) 30 minutes before bilateral intra-plantar c48/80 injection (1.5μg/paw; 10μl). 2.5h after c48/80 treatment, their hind paws were excised and preserved for TNF-α protein quantification by ELISA ( **A**). Another set of mice was euthanized at 3h after c48/80 treatment; their footpads were excised for TNF-α mRNA abundance quantification by qRT-PCR ( **B**). Bars represent average pg/ml TNF-α protein ( **A**) and fold-expression of TNF-α transcripts compared to β2m ( **B**) and the error bars represent ±SEM. n = 6 (Sal/Sal); 9 (Sal/c48/80) for ELISA assays and n = 3 mice per treatment group for qRT-PCR; data are representative of 3 (ELISA) and 2 (qPCR) experiments.

Mast cells contain pre-formed TNF-α in their granules ^16^ that is released early upon degranulation ^16^. We analyzed TNF-α protein and mRNA levels in c48/80-treated and untreated hind paw tissue to determine whether there was new synthesis of TNF-α immediately following treatment with the basic mast cell secretagogue. We found that neither protein nor mRNA levels of the cytokine detectably increased in the tissue within 3 hours following intra-plantar c48/80 injection ( Figure 3A–B). Thus, it is most likely that the anti-TNF-α neutralizing antibody treatment targets pre-formed, rather than newly synthesized, TNF-α molecules and this blockade contributes to the resulting decrease in thermal sensitivity in the hind paw tissue.

### TNF-α neutralizing antibody pre-treatment does not affect early neutrophil recruitment in the tissue

**Figure 4.**
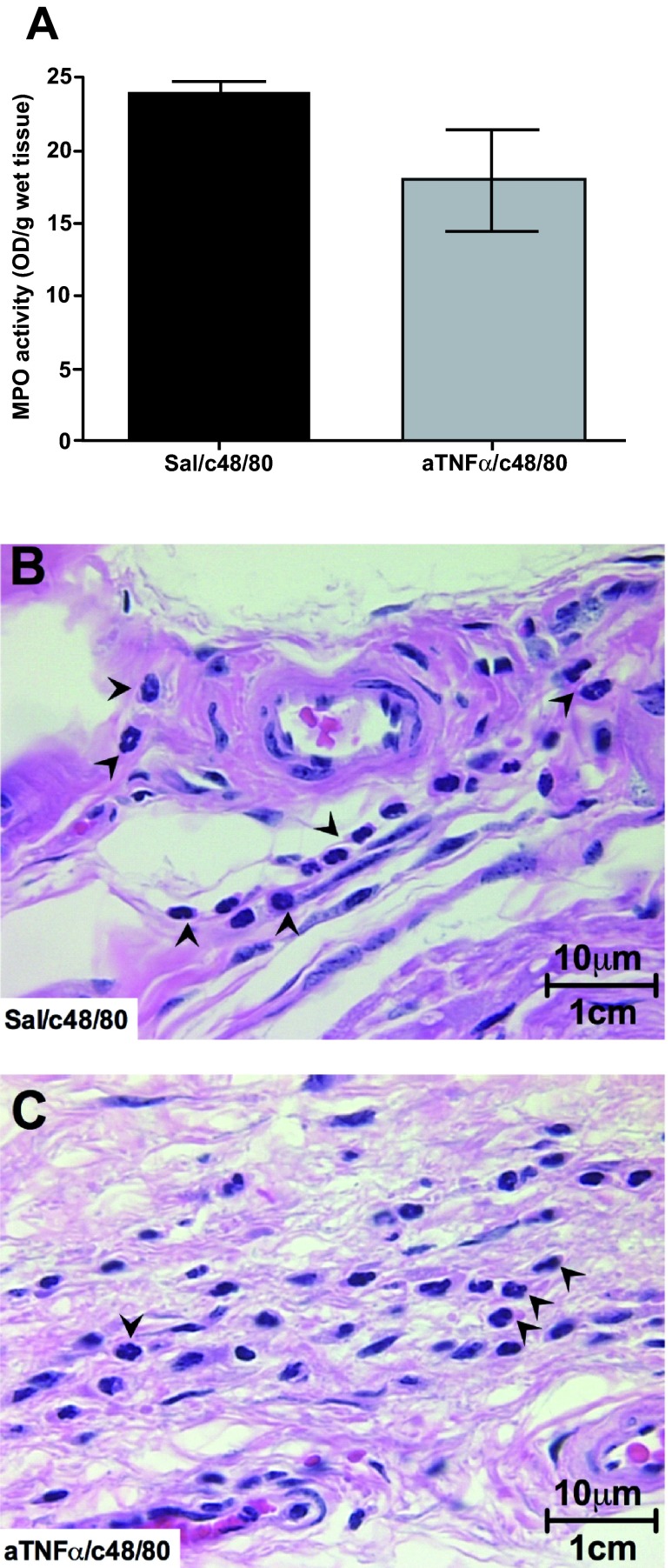
Pre-treatment with anti-TNF-α does not prevent c48/80-provoked neutrophil influx. Mice that received c48/80 intra-plantar injections following anti-TNF-α pretreatment did not have significantly different levels of myeloperoxidase enzyme activity compared to mice that received vehicle pretreatment ( **A**). Hind paws were harvested from euthanized mice and assayed for MPO activity; bars represent average MPO activity as OD/g wet tissue and error bars represent ±SEM ( **A**). Mice were euthanized 2.5h after c48/80 administration, their hind paws were harvested, preserved in 10% buffered formalin and ethanol, and 4μm paraffin sections stained with hematoxylin & eosin for neutrophil visualization at 1000x. Similar to c48/80-treated mice ( **B**), mice pre-treated with anti-TNF-α neutralizing antibody showed clear evidence of neutrophil influx (indicated by black arrowheads) into the affected hind paw tissue ( **C**). n = 3 mice per treatment group for MPO assay; data are representative of 2 experiments.

Neutrophil influx into the affected tissue contributes to c48/80-provoked hind paw thermal sensitivity ^2^ and TNF-α is a known neutrophil attractant ^3, 17^. We have previously shown that the blockade of neutrophil influx can abrogate mast cell-dependent thermal sensitivity in the mouse hind paw ^2^. Therefore, we investigated whether pre-treatment with the TNF-α neutralizing antibody had an effect on neutrophil influx. We found that levels of myeloperoxidase (an enzyme indicating the presence of activated neutrophils) in the hind paw tissue of mice injected bilaterally with 1500ng of c48/80 were not significantly reduced with anti-TNF-α pre-treatment ( Figure 4A). We further confirmed this by examining the presence of neutrophils in 4μm paraffin embedded hind paw tissue sections stained with H&E and found that infiltrating neutrophils were present in the hind paws of mice that received TNF-α neutralizing antibody ( Figure 4B). Neutrophil numbers in the hind paw tissue of mice following c48/80 injection with and without anti-TNF-α pre-treatment were approximately a total of ~150 neutrophils in 10 randomly chosen visualized sections per paw (see Data File). Therefore, pre-treatment with anti-TNF-α neutralizing antibody had little to no inhibitory effect on the levels of tissue infiltrating neutrophils and myeloperoxidase enzyme activity in the hind paw tissue of c48/80-treated mice.

Effect of TNF-α neutralizing antibody on thermal sensitivity qPCR: The data are cycle threshold (CT) values of beta2m (reference) and TNF-alpha.
Neutrophil counts: Neutrophils were counted in the plantar region from heel to the toes, in 10 separate sections (fields of view) at 1000x. Total number of neutrophils equals the sum of neutrophils in all 10 sections.
Myeloperoxidase activity: The data show optical density (OD) readings at 450nm and "BA" values = OD - average of the blanks. Average "BA" values per gram of tissue weight = MPO activity as OD/gm of wet tissue.
Mast cell degranulation: The data show numbers and percentages of intact and mildly, moderately, extensively degranulated mast cells in the hind paw tissue.
Hyperalgesia: "BL Latency" signifies baseline latency values in seconds and "Delta latency" signifies change in thermal latency (experimental average - baseline average) at each time point for each mouse.
Edema: "BL R" and "BL L" = baseline paw width in mm for the right and left hindpaw of each mouse respectively. Baseline values are subtracted from averages of left (L) and right (R) paw widths at each time point to give the delta paw width at each time point.
ELISA: The data show the concentrations of TNF-alpha protein in hind paw tissue lysate assayed in duplicate and the average concentration in pg/ml of paw lysate.

## Discussion

Pain is a complex physiological and pathological phenomenon with intricate underlying neuro-immune circuitry. Pain can be either protective or maladaptive ^1, 11^ and in the former instance, serves as an early warning system that prompts an organism to retreat from noxious environmental stimuli.

We have previously shown that mast cells are required for enhanced sensitivity to heat stimulus following c48/80 treatment in the hind paw tissue of mice and blockade of neutrophil influx or histamine signaling can abrogate these responses ^2^. Here we show that pre-treatment with TNF-α neutralizing antibody just prior to c48/80 administration significantly reduces early thermal hyperalgesic responses. TNF-α neutralizing antibody treatment does not affect mast cell degranulation; levels of mild, modest and extensive degranulation following c48/80 treatment were similar to those previously described ^2^. Mast cells contain pre-formed TNF-α in their cytoplasmic granules and this pro-inflammatory cytokine is one of the earliest mediators released by these cells upon activation ^16^. We looked for evidence of changes in TNF-α protein and mRNA levels shortly after c48/80 treatment and found none, suggesting that the pre-formed TNF released by mast cells may be the main source of this cytokine in these experiments. As TNF-α is a known attractant of neutrophils ^17^, we expected TNF-α blockade to result in a reduction of infiltrating neutrophils. We have previously shown that blockade of neutrophil influx can abrogate c48/80-provoked thermal sensitivity ^2^. However, we found here that in the first 2.5 hours following c48/80 administration, TNF-α neutralizing antibody pre-treatment had little to no effect on recruitment of tissue neutrophils. The influx of neutrophils in the presence of TNF-α neutralizing antibodies is supported by several different kinds of evidence – myeloperoxidase activity in the tissue, visualization of neutrophils by H&E staining and neutrophil counts in H&E stained tissue sections. However, given our relatively small sample size for these assays, it is possible that a reduction in neutrophil influx can be seen with larger numbers of mice. Nevertheless, our observations are in keeping with earlier studies that show that mast cell mediators other than TNF-α can contribute to the induction of neutrophil influx ^18^. In these experiments, pain was significantly abrogated as early as 30 minutes after c48/80 treatment despite the presence of neutrophils.

TNF-α is one of the early inflammatory cytokines with a known role in various nociceptive cascades ^6, 7^. Direct injection of TNF-α in the hind paw tissue produces thermal and mechanical hyperalgesia in rodents ^6, 19^; anti-TNF-α antibody administration shortly before challenge reduces the hyperalgesic responses to carrageenan ^12^ in mice and blockade of TNF-α induced neutrophil influx impairs thermal carrageenan pain in rats ^3^.

Our findings here add nuance to the prevailing model of mast cell and neutrophil modulation of peripheral nociception with evidence that TNF-α blockade can also abrogate very early thermal pain responses without a proportional reduction in neutrophil influx. Hind paw thermal pain provoked by c48/80 is markedly abrogated despite the presence of infiltrating neutrophils in the tissue. In a model of carrageenan-induced pain in rats, Cunha and colleagues have shown that levels of TNF-α in the tissue are not affected by the absence of infiltrating neutrophils i.e. these cells are not the primary sources of TNF-α ^3^ but rather the release of TNF-α and other cytokines such as IL-1β in the tissue following an inflammatory insult serves to attract infiltrating neutrophils that continue to fuel the nociceptive cascade ^3^. In preliminary experiments, we have found that pre-treatment with antibodies against IL-1β reduces neutrophil influx into the tissue but does not abrogate c48/80-provoked thermal pain at early time points (data not shown). Therefore, the rapid anti-hyperalgesic effect that we observe here may be due to the blockade of earlier effects of TNF-α on nociceptor sensitization mediated by cyclooxygenase (COX) and p38 mitogen-activated protein (MAP) kinase activation ^9, 20^ or effects of TNF-α on nociceptor ion channels ^10, 21^. Our observations here suggest that pre-formed TNF-α, e.g. from mast cell granules in the skin mast cells, is the likely target of the TNF-α neutralizing antibody that produces an early reduction of thermal sensitivity. This is underscored by the documented proximity of nerves and mast cells in the tissue ^8^ and the known effects of mast cell-derived TNF-α on nerve physiology - elongation of cutaneous nerves in oxazolone contact hypersensitivity ^22^, and susceptibility of sensory neurons to agents such as capsaicin, in a model of tracheal vascular hyper-permeability ^23^. These known interactions further support the idea that nociceptive neurons could be early downstream targets of the first release of pre-formed TNF-α immediately after mast cell degranulation. In this study, we show that early, protective, nociceptive withdrawal responses to heat stimulus are compromised by the blockade of TNF-α signaling. It has been documented that individuals who use TNF-targeted biological therapeutics to manage chronic autoimmune and inflammatory conditions can experience a higher risk of bacterial and viral infections ^24^. Our findings here suggest that the use of TNF-α blockade therapies may also compromise the protective, nociceptive responses of such individuals leaving them more vulnerable to tissue damage from injurious environmental stimuli.
