# Supplementary material for: TNF-alpha neutralizing antibody blocks thermal sensitivity induced by compound 48/80-provoked mast cell degranulation
Source: F1000Res. 2013 Sep 27;2:178. Originally published 2013 Aug 21. [Version 2] doi: 10.12688/f1000research.2-178.v2 (PMC3869523; doi:10.12688/f1000research.2-178.v2)
Supplement: Peer review history

# Peer review: Referee response for version 2

**Published:** 2013-10-07

**Authors:** Adrian Piliponsky

**Affiliations:** 1 Seattle Children's Hospital, Seattle, USA

Thank you for addressing my comments.

I have read this submission. I believe that I have an appropriate level of expertise to confirm that it is of an acceptable scientific standard.

# Peer review: Referee response for version 2

**Published:** 2013-09-30

**Authors:** Rosa Torres

**Affiliations:** 1 Department of Pharmacology of Therapeutics and Toxicology, Autonomous University of Barcelona, Barcelona, Spain

The paper is now more clear, and has improved after addressing the referees' comments.

Thank you and congratulations to the authors.

I have read this submission. I believe that I have an appropriate level of expertise to confirm that it is of an acceptable scientific standard.

# Peer review: Referee response for version 2

**Published:** 2013-09-30

**Authors:** Peter Drummond

**Affiliations:** 1 Murdoch University, Perth, Australia

Thank you for addressing my previous comments. This paper makes an important contribution to the literature.

I have read this submission. I believe that I have an appropriate level of expertise to confirm that it is of an acceptable scientific standard.

# Peer review: Referee response for version 1

**Published:** 2013-09-02

**Authors:** Rosa Torres

**Affiliations:** 1 Department of Pharmacology of Therapeutics and Toxicology, Autonomous University of Barcelona, Barcelona, Spain

Chatterjea *et al.* show that TNF-alpha blockade reduced early tissue swelling in a mast cell-dependent thermal hyperalgesia model. The paper is well constructed and clear, and experiments have been well designed and conducted. There are minor questions that I mention below.

1. Anti-TNF-a neutralizing antibody dose is mainly expressed in the article as ul/Kg, please write it as ug/Kg.
2. Since toluidine blue staining is performed differently in different tissues please indicate pH and the time of incubation.
3. In the Methods for TNF-a expression measurements it is said “relative transcript abundance was determined by quantitative RT-PCR” but the technique used is semi-quantitative.
4. When explaining Figure 1A does not mention that at 30 minutes it is also significantly different when comparing the control and TNF treated groups.
5. Please comment more on the mast cells degranulation status of figure 2. Is 60% mild degranulation for this model an expected value? Have you seen or would you expect different percentages using different C4880 doses?
6. Pre-treatment with TNF-a antibody seems to have a small effect on MPO activity but not a significant one, probably because of the low n number (n = 3). This could be discussed as a possibility.

I have read this submission. I believe that I have an appropriate level of expertise to confirm that it is of an acceptable scientific standard.

# Response

**Published:** 2013-09-24

**Authors:** Devavani Chatterjea

**Affiliations:** Macalester College, USA

Dear Dr. Torres,

Thank you for reviewing our paper.  Our responses to your comments follow:

 

1. We have now corrected this throughout the manuscript.

2. We have added this information to the relevant methods section.

3. We have clarified that our approach uses comparative quantitative PCR analysis and added an additional reference for this nomenclature.  

4. We have added this to the description of the report of the results from Figure 1A.

5. We have added a sentence to the discussion indicating that the levels of degranulation seen here are similar to those we have previously observed.

6. We have added some clarifying language indicating that these findings are based on a relatively small sample size but that multiple lines of evidence indicate that at least in these experiments, treatment with anti-TNF neutralizing antibodies did not completely abrogate neutrophil influx into the c48/80-treated hind paw tissue.

# Peer review: Referee response for version 1

**Published:** 2013-08-29

**Authors:** Adrian Piliponsky

**Affiliations:** 1 Seattle Children's Hospital, Seattle, USA

The study by Chatterjea D *et al*. is well-designed; its conclusions are balanced and justified based on the results shown. I think that it could be relevant to know whether TNF-alpha injections can mimic the effects of c48/80 injections in the model used: hyperalgesic withdrawal responses.

Figure 4a leads to the conclusion that neutrophil influx is not affected by treatment with anti-TNFalpha antibodies and c48/80. Data is based on n=3 according to figure legend, though there is a trend for neutrophil numbers to be reduced by treatment with anti-TNFalpha antibodies. I am wondering whether the authors have considered increasing the number of mice for this experiment. If it is definitely not the case that neutrophils numbers are reduced, it remains unexplained why there is a discrepancy with a previous study, in which the authors demonstrated that neutrophils are an important component of the hyperalgesic response induced by c48/80.

I have read this submission. I believe that I have an appropriate level of expertise to confirm that it is of an acceptable scientific standard.

# Response

**Published:** 2013-09-24

**Authors:** Devavani Chatterjea

**Affiliations:** Macalester College, USA

Dear Dr. Piliponsky,

Thank you for reviewing our paper. 

In our discussion of the finding in Figure 4A, we have now added some clarifying language indicating that these findings are based on a relatively small sample size but that multiple lines of evidence indicate that at least in these experiments, treatment with anti-TNF neutralizing antibodies did not completely abrogate neutrophil influx into the c48/80-treated hind paw tissue.

# Peer review: Referee response for version 1

**Published:** 2013-08-29

**Authors:** Peter Drummond

**Affiliations:** 1 Murdoch University, Perth, Australia

This paper makes a strong case for the early involvement of mast cell-derived TNF-alpha in thermal hyperalgesia and oedema during inflammatory reactions. The only contentious issue is the suggestion that TNF-alpha acts directly on nociceptors, rather than via the cascade of inflammatory mediators triggered by TNF release. The references cited in support of this view either did not directly address this point, or did not provide the intended support. For example, Zhang *et al*. (2011) (reference 9 in the paper) proposed that TNF-alpha sensitizes meningeal nociceptors indirectly via local COX activity and the p38 MAPK signalling cascade, rather than by direct actions on nociceptors. Wang *et al*. (J Mol Neurosci 2007;32:207-216) reported that TNF receptors in the sciatic nerve were expressed primarily on Schwann cells rather than neurons, leaving open the possibility that Schwann cells are involved in the inflammatory cascade triggered by TNF-alpha.

One other minor point: I think this sentence in the first paragraph of the Results "Saline-treated control mice had no increase in thermal sensitivity and showed no tissue swelling after c48/80 treatment" should be "Saline-treated control mice had no increase in thermal sensitivity and showed no tissue swelling".

I have read this submission. I believe that I have an appropriate level of expertise to confirm that it is of an acceptable scientific standard.

# Response

**Published:** 2013-09-24

**Authors:** Devavani Chatterjea

**Affiliations:** Macalester College, USA

Dear Dr. Drummond,

Thank you for reviewing our paper. 

Following your suggestion, we have rephrased the sentence describing the effects of TNF-a on nociceptors as possibly mediated by inflammatory intermediates. We have also corrected the sentence in the first paragraph of the results section.
